# Ajuforrestin A, an Abietane Diterpenoid from *Ajuga ovalifolia* var. *calanthe*, Induces A549 Cell Apoptosis by Targeting SHP2

**DOI:** 10.3390/molecules27175469

**Published:** 2022-08-25

**Authors:** Hongling Yan, Miao Jiang, Fujin Yang, Xueyong Tang, Mao Lin, Chunyan Zhou, Yuzhu Tan, Deming Liu

**Affiliations:** 1 Key Laboratory of Southwestern Chinese Medicine Resources, Pharmacy College, Chengdu University of Traditional Chinese Medicine, Chengdu 611137, China; 2 Chongqing Clinical Research Center for Dermatology, Department of Dermatology, Chongqing Traditional Chinese Medicine Hospital, Chongqing 400011, China; 3 Chongqing Key Laboratory of Integrative Dermatology Research, Department of Dermatology, Chongqing Traditional Chinese Medicine Hospital, Chongqing 400011, China; 4 Key Laboratory of External Therapies of Traditional Chinese Medicine in Eczema, Department of Dermatology, Chongqing Traditional Chinese Medicine Hospital, Chongqing 400011, China; 5 General Surgery, Chongqing Traditional Chinese Medicine Hospital, Chongqing 400021, China

**Keywords:** SHP2, Ajuforrestin A, *Ajuga ovalifolia* var. *calantha*, apoptosis, ERK/AKT pathway

## Abstract

The Src-homology 2 domain-containing phosphatase 2 (SHP2), which is encoded by PTPN11, participates in many cellular signaling pathways and is closely related to various tumorigenesis. Inhibition of the abnormal activity of SHP2 by small molecules is an important part of cancer treatment. Here, three abietane diterpenoids, named compounds **1**–**3**, were isolated from *Ajuga ovalifolia* var. *calantha*. Spectroscopic analysis was used to identify the exact structure of the compounds. The enzymatic kinetic experiment and the cellular thermal shift assay showed compound **2** selectively inhibited SHP2 activity in vitro. Molecular docking indicated compound **2** targeted the SHP2 catalytic domain. The predicted pharmacokinetic properties by SwissADME revealed that compound **2** passed the majority of the parameters of common drug discovery rules. Compound **2** restrained A549 proliferation (IC_50_ = 8.68 ± 0.96 μM), invasion and caused A549 cell apoptosis by inhibiting the SHP2–ERK/AKT signaling pathway. Finally, compound **2** (Ajuforrestin A) is a potent and efficacious SHP2 inhibitor and may be a promising compound for human lung epithelial cancer treatment.

## 1. Introduction

Protein tyrosine phosphatases (PTPs) control various cellular processes, regulate the tyrosine phosphorylation process with protein tyrosine kinases (PTKs) and control key signal transduction [1]. Dysfunction of tyrosine phosphorylation is associated with tumors, obesity, and immune diseases, therefore, the inhibition of PTPs and PTKs is important for disease treatment [2]. Increasing evidence has indicated that SHP2, PTP1B and CDC25, which belong to the PTP family, can enhance signal transduction and are potential oncogenes [3,4]. Those PTPs are promising targets for cancer treatment, and specific PTP small molecule inhibitors have been attracting increasing attention in recent years [5]. SHP2, which is encoded by PTPN11, is widely expressed in human tissues. Src-homology (SH) 2 and a catalytic (PTP) domain constitute SHP2. The two SH domains act as the phosphor-tyrosine binding sites for SHP2 substrates, and the PTP region contains significant biological activity [6]. The intramolecular interaction between the N-SH2 and PTP domains impedes substrates’ access and results in SHP2 in a self-inhibited state. Numerous research has indicated that SHP2 is an essential transducer of cellular cytokine, growth factor, and controls T-cell activation, proliferation, and apoptosis. As it has a key role in the RAS/MAPK and JAK/STAT signaling pathways, SHP2 is regarded as an important oncogene for several leukemias and some tumor processes. Gain of function mutations cause the abnormal activation of SHP2 phosphatase and are closely related to the incidence of blood-system tumors, pancreatic cancer and non-small cell cancers. Many types of research have already revealed that 35% of juvenile myelomonocytic leukemia (JMML) and 50% of Noonan syndrome patients result from SHP2 mutants [7]. The majority of mutants in SHP2 enhance the PTP domain catalytic activity; those mutants attenuate the auto-inhibition state. More and more evidence suggesting the important role of SHP2 in disease development indicates that natural SHP2 inhibitors are essential for therapy [8,9]. As SHP1 and SHP2 share 75% sequence in PTP domains, it is difficult to develop new selectivity SHP2 inhibitors [10].

*Ajuga ovalifolia* var. *calantha*, a plant of the genus Ajuga (Labiatae), is a folk medicine for the treatment of fever, toothache, dysentery, hypertension, diabetes, gastrointestinal disorders, malaria, and also has antibacterial, anti-fungal, anti-inflammatory, anti-tumor and insect antifeeding properties. In previous work, we found that abietane diterpenoid isolated from *Ajuga ovalifolia* var. *calantha* obvious inhibited A549 cell growth [11,12]. As a continuous work for the discovery of bioactive natural SHP2 inhibitors in cancer therapy, an abietane diterpenoid, compound **2** (Ajuforrestin A), was studied in this research, which was better than our previous reported compound 3-acetoxylteuvincene G (3-AG). Herein, we explain in detail its isolation, structure identification, inhibition of SHP2 or SHP1, and the potential mechanism of compound **2** inducing A549 apoptosis.

## 2. Results

### 2.1. Phytochemical Investigation

**Figure 1:**
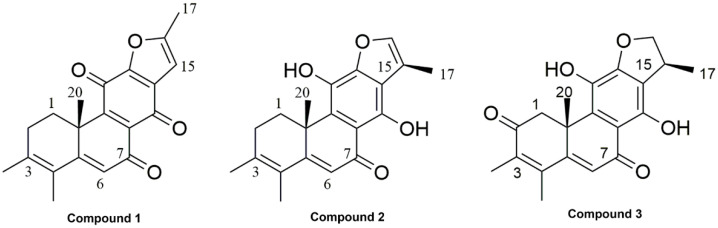
The structure of compounds **1**–**3**. (compound **1**, 12, 16-epoxy-17(15→16), 18(4→3)-diabeo-abieta-3, 5, 8, 12, 15-pentaene-7, 11, 14-trione; compound **2**, Ajuforrestin A; compound **3**, Ajudecumin A).

12, 16-epoxy-17(15→16), 18(4→3)-diabeo-abieta-3, 5, 8, 12, 15-pentaene-7, 11, 14-trione (compound **1**, Figure 1): Orange powder; ^1^H NMR (400 MHz, CDCl_3_) δ: 6.60 (1H, s, H-15), 6.28 (1H, s, H-6), 3.31 (1H, dd, J = 13.3, 5.7 Hz, H-1b), 2.55 (1H, m, H-2b), 2.46 (3H, s, H-17), 2.24 (1H, dd, J = 19.4, 5.1 Hz, H-2a), 1.93 (3H, s, H-19), 1.90 (3H, s, H-18), 1.64 (1H, td, J = 13.3, 5.8 Hz, H-1a), 1.54 (3H, s, H-20); ^13^C NMR (100 MHz, CDCl_3_) δ: 183.9 (C-7), 180.8 (C-11), 175.2 (C-14), 163.1 (C-5), 160.9 (C-12), 155.8 (C-9), 150.2 (C-16), 141.2 (C-3), 131.2 (C-13), 129.7 (C-8), 124.9 (C-4), 122.3 (C-6), 104.6 (C-15), 40.6 (C-10), 31.1 (C-2), 29.9 (C-1), 24.4 (C-17), 20.9 (C-20), 14.9 (C-19), 14.3 (C-18) (The MS and NMR spectra shown in Figures S1–S3) [13].

Ajuforrestin A (compound **2**, Figure 1): Orange powder, HRESIMS m/z 347.1247 [M+Na]^+^ (calcd for C_20_H_20_O_4_Na^+^, 347.1254). ^1^H NMR (400 MHz, Acetone-d_6_) δ: 14.32 (1H, s, 14-OH), 8.36 (1H, brs, 11-OH), 7.52 (1H, s, H-16), 6.22 (1H, s, H-6), 3.50 (1H, dd, J = 13.3, 4.5 Hz, H-1b), 2.58 (1H, br t, H-2b), 2.38 (3H, d, J = 1.4 Hz, H-17), 2.28 (1H, dd, J = 18.7, 5.5 Hz, H-2a), 1.96 (3H, s, H-18), 1.92 (3H, s, H-19), 1.62 (1H, m, H-1a), 1.58 (3H, s, H-20); ^13^C NMR (100 MHz, Acetone-d_6_) δ: 192.1 (C-7), 167.1 (C-9), 154.5 (C-12), 151.4 (C-11), 142.0 (C-3), 141.9 (C-16), 133.5 (C-14), 131.8 (C-8), 125.9 (C-4), 118.9 (C-6), 118.1 (C-15), 117.3 (C-13), 110.0 (C-5), 40.5 (C-10), 30.9 (C-2), 30.5 (C-1), 22.7 (C-20), 20.7 (C-19), 15.2 (C-18), 9.6 (C-17) (The NMR spectra shown in Figures S4 and S5) [14].

Ajudecumin A (compound **3**, Figure 1): Orange powder, HRESIMS m/z 363.1214 [M+Na]^+^ (calcd for C_20_H_20_O_5_Na^+^, 363.1203), ^1^H NMR (400 MHz, CDCl_3_) δ: 13.29 (1H, br.s, 14-OH), 6.52 (1H, s, H-6), 5.39 (1H, brs, 11-OH), 4.81 (1H, dd, J = 9.2, 8.7 Hz, H-16b), 4.32 (1H, dd, J = 8.7, 5.8 Hz, H-16a), 4.18 (1H, d, J = 16.7 Hz, H-1b), 3.76 (1H, m, H-15), 2.43 (1H, d, J = 16.9 Hz, H-1a), 2.22 (3H, s, H-19), 2.00 (3H, s, H-18), 1.63 (3H, s, H-20), 1.43 (3H, d, J = 6.9 Hz, H-17); ^13^C NMR (100 MHz, CDCl_3_) δ: 197.4 (C-2), 189.1 (C-7), 160.8 (C-5), 155.3 (C-14), 154.9 (C-12), 146.3 (C-4), 136.3 (C-3), 134.5 (C-9), 131.4 (C-11), 124.1 (C-6), 116.8 (C-13), 109.2 (C-8), 81.2 (C-16), 45.6 (C-1), 42.8 (C-10), 35.8 (C-15), 25.0 (C-20), 18.5 (C-17), 17.5 (C-19), 12.1 (C-18) (The MS and NMR spectra shown in Figures S6–S8) [15].

### 2.2. Compound ***2*** Inhibits A549 Cell Proliferation Targeting SHP2

**Figure 2:**
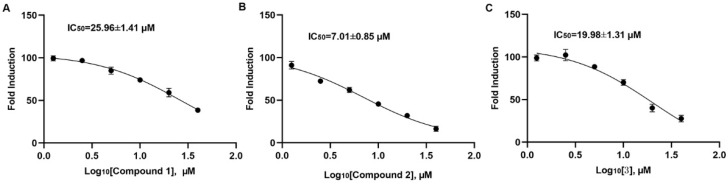

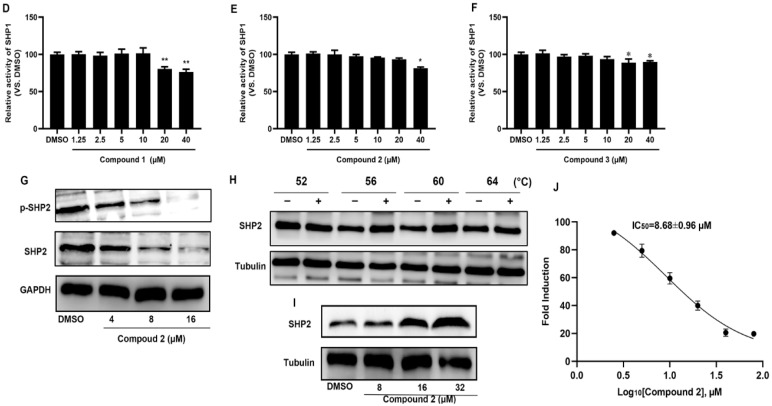
The inhibitory effects of compounds **1**–**3** on SHP1 and SHP2. The IC_50_ of compounds **1**–**3** (**A**–**C**) on SHP2. The inhibitory effects of compounds **1**–**3** (**D**–**F**) on SHP1. (**G**) The effects of compound **2** on cellular SHP2 expression. (**H**,**I**) Cellular thermal shift assay between SHP2 and compound **2**. (**J**) The cytotoxicity of compound **2** in A549 cells. Cont., DMSO control. (*) *p* < 0.05, (**) *p* < 0.01 compared with the control group.

For the discovery of an SHP1 and SHP2 natural small molecules inhibitor, we purified SHP1 and SHP2 protein and constructed the screening system. Figure 2A–C show that compounds **1**–**3** can inhibit SHP2 catalytic activity with IC_50_ of 25.96 ± 1.41 μM, 7.01 ± 0.85 μM, and 19.98 ± 1.31 μM, respectively. At the same time, we also observed that compound **2** exerted no inhibitory effects on SHP1 below 20 μM and compounds **1** and **3** inhibited SHP1 catalytic activity at high concentrations (Figure 2D–F). Those enzymatic experiments indicated that compound **2** may directly inhibit SHP2 catalytic activity in vitro. Furthermore, the effects of compound **2** on A549 cellular SHP2 were also investigated. After 72 h of treatment with compound **2**, we found that compound **2** suppressed SHP2 expression in a dose-dependent manner (Figure 2G and Figure S10). To further demonstrate that compound **2** directly targeted SHP2, we performed cellular thermal shift experiments (CETSA). The CETAS results revealed that the decrease in SHP2 expression with increasing temperature was suppressed with various concentration compound **2** treatments (Figure 2H and Figure S10). In addition, the increase in the concentration of compound **2** also raised SHP2 stability (Figure 2I). The CCK-8 results showed that compound **2** obviously inhibited A549 cell proliferation with an IC_50_ of 8.68 ± 0.96 μM (Figure 2J). In summary, our experiments confirmed the interaction between compound **2** and SHP2 resulted in the inhibition of A549 cell proliferation.

### 2.3. Molecular Docking of Compound ***2*** with SHP2

**Figure 3:**
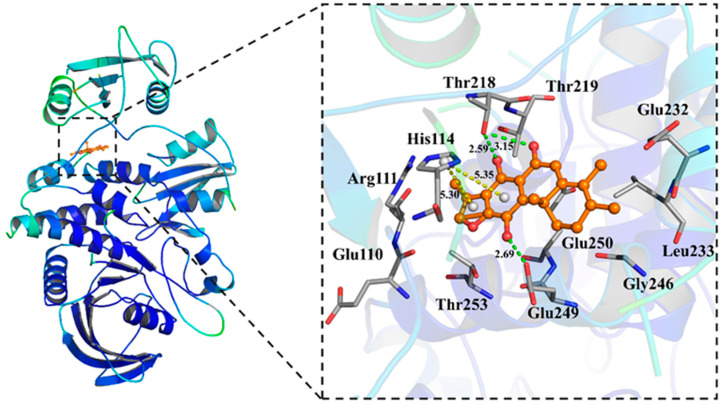
Molecular docking of compound **2** (B) with SHP2 (PDB ID: 5EHR, green dash, hydrogen bonds; yellow dash, π–cation interaction; other residues, hydrophobic interactions).

To assess the binding sites of compound **2**, computer docking using a published SHP2 structure (PDB ID: 5EHR) was conducted. Among the conformers generated by AutoDock Vina 1.1.2, (version 1.1.2, Molecular Graphics Lab, CA, USA), we predicted that the ligand would be oriented to the bind pose, as shown in Figure 3, which has the lowest affinity energy (−7.8 Kcal/mol) and RMSD value (Figures S11 and S12). The in silico analysis of compound **2** showed that it formed favorable hydrogen bonds with Thr218 and Glu249 (Figure 3). Additionally, compound **2** also formed a π–cation interaction with His114 and hydrophobic interactions with Clu110, Arg111, Glu249, Gly246, Leu233, Glu250, Glu232, Thr219 and Thr253 (Figure 3, Figures S13 and S14 and Table S1). The docking suggested that compound **2** participates in stable contact with the allosteric domain of SHP2 and the binding site onto SHP2 would be similar to that of SHP099 (Figures S10, S15 and S16 and Table S1).

### 2.4. Pharmacokinetic Properties Prediction of Compound ***2***

Predicting the pharmacokinetic properties is beneficial for understanding and predicting the biological action of drugs, such as toxicity or therapeutic effects [16]. Here, we performed in silico physicochemical predictions using the SwissADME platform. The pharmacokinetic properties of compound **2**, including the pharmacokinetic properties, lipophilicity, water solubility, drug-likeness, and medicinal chemistry were identified and listed in Supplementary Table S2. Firstly, compound **2** had a topological polar surface area (TPSA) of 70.67 Å2, which revealed that it could permeate cell membranes [17]. Simultaneously, the consensus lipophilicity (Log Po/w) of compound **2** was 3.74 which demonstrated good lipophilicity. Next, the water solubility (Log S) analysis revealed that it had moderate water solubility. Pharmacokinetic data showed that compound **2** had high GI absorption (gastrointestinal absorption) and could permeate the BBB (blood–brain barrier). According to the above information, it could be inferred that the compound was suitable for oral administration. Finally, compound **2** obeyed Lipinski’s rule of five. Together, the results suggested that compound **2** was effective and druggable in the study.

### 2.5. Compound ***2*** Attenuates A549 Invasion and Migration by the Inhibition of the ERK, AKT Pathway

**Figure 4:**
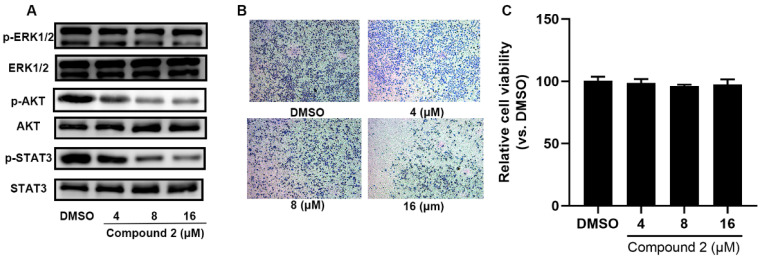
Compound **2** restrains RAF–MEK–ERK signal pathway’s abnormal activation by the down-regulation of SHP2 phosphatase. (**A**) The effects of 72 h of compound **2** treatment on ERK, AKT signaling pathway in A549 cell. (**B**) The effects of 24 h of compound **2** treatment on A549 cell invasion. (**C**) After treatment with DMSO or compound **2** for 24 h, cell viability of A549 cells was detected.

**Figure 5:**
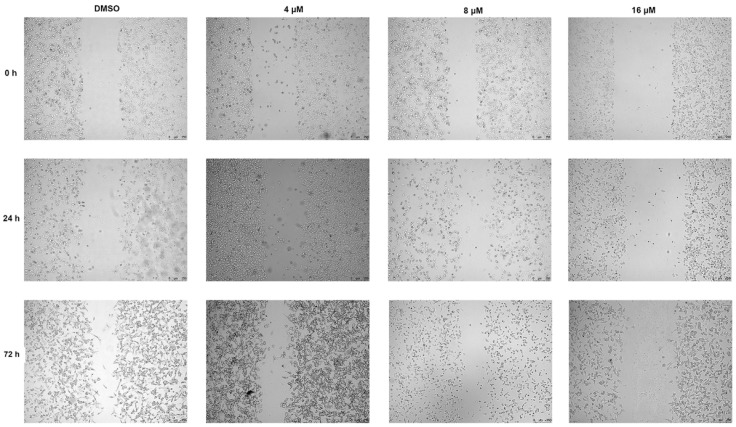
The effects of compound **2** on A549 cell migration.

It was reported that SHP2 phosphorylation activates the RAF–MEK–ERK signaling pathway and causes precancerous lesion formation and tumorigenesis [16,17]. As shown in Figure 4A, compound **2** markedly inhibited p-ERK1/2 expression. The PI3K-AKT and STAT3 signaling pathways, the effectors of RAS, were obviously inhibited after 72 h treatment with various concentrations of compound **2** (Figure 4A and Figure S17). Furthermore, we also observed that A549 cell invasion ability obviously decreased after 24 h incubation with compound **2** in a dose-dependent manner and treatment with compound **2** for 24 h did not affect A549 cell viability (Figure 4B,C). As shown in Figure 5, a wound-healing assay was performed to detect the effect of compound **2** on A549 cell migration. The result showed that treatment with compound **2** significantly restrained A549 cell migration. In all, the down-regulation of SHP2 phosphatase by compound **2** restrains the RAF–MEK–ERK signaling pathway’s abnormal activation.

### 2.6. Compound ***2*** Causes A549 Cell Apoptosis

**Figure 6:**
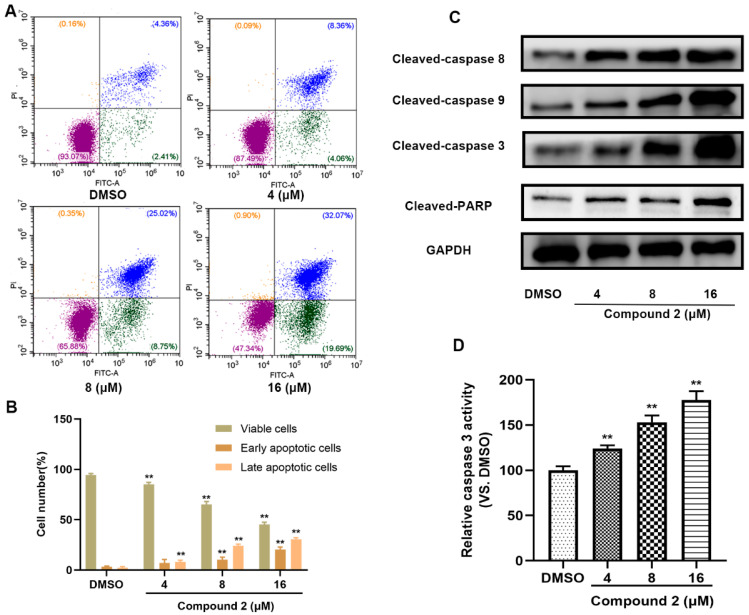
Compound **2** causes A549 cell apoptosis. (**A**) After 72 h of corresponding compound **2** treatment, A549 cells were collected and the apoptotic cell was detected by flow cytometry. (**B**) The percentage of apoptotic cells induced by compound **2**. (**C**) The effects of a 72 h compound **2** treatment on apoptosis-related signaling pathway. (**D**) After compound **2** treatment for 72 h, caspase-3 activity was detected. (**) *p* < 0.01 compared with the control group. Cont., DMSO control.

To further reveal the underlying mechanism of compound **2** causing A549 cytotoxicity, an apoptosis assay was performed. As depicted in Figure 6A,B, an obvious increase in apoptotic cells was detected after incubation with compound **2** for 72 h. In addition, cleaved caspase-3, 8, 9, which were closely related to apoptosis, were up-regulated after various doses of the treatment with compound **2**, and we also observed the cleaved-PARP were also activated (Figure 6C and Figure S18). Finally, compound **2** incubation led to a significant rise in caspase-3 expression in a dose-dependent manner (Figure 6D). Those results revealed that the inhibition of SHP2 by compound **2** eventually results in a caspase-3 medicated apoptosis signaling pathway.

## 3. Discussion

SHP2 up-regulation is observed in lots of human diseases, such as solid tumors, hematologic malignancies and immune diseases [18,19]. It was found that the restrain of SHP2 catalytic activity can regulate cancer cell proliferation and is a potential target for tumor therapy. In this research, compounds **1**–**3**, three abiterpenoids, were extracted from *Ajuga ovalifolia* var. *calantha* and their exact structures were identified by extensive spectroscopic analysis. We also discovered that compound **2** can directly target SHP2 and inhibit its catalytic activity and its inhibitory effects were better than compounds **1** and **3**, but an inhibitory effect of compound **2** on SHP1 was not observed. In addition, the expression of SHP2 and phosphorylation of SHP2 were down-regulated after incubation with compound **2** for 72 h in A549 cells. Molecular docking shows that compound **2** may interact with SHP2 by the formation of hydrogen bonds, π–cation interaction and hydrophobic interactions. The predicted pharmacokinetic properties by SwissADME revealed that compound **2** passed the majority of parameters of common drug discovery rules. SHP099, a well know small inhibitor, formed stable hydrogen bonds with Thr218 and Glu249, and had hydrophobic interactions with the residues Arg111, Gly246, Leu233, Glu249, Glu232, Thr219 and Thr253 with an affinity of -10.9 kcal/mol, and these bonding sites were similar to compound **2**. In addition, the enzymatic assays, CETSA and docking experiments confirmed that compound **2** induced A549 cell apoptosis by directly targeting SHP2 phosphatase through hydrogen bonds and hydrophobic interactions between SHP2 and compound **2**. SHP2 catalytic activity is essential for the full activation of the RAS–RAF–MAPK pathway. Our results showed that compound 2 can significantly inhibited the phosphorylation of ERK. The PI3K/ATK pathway, an important SHP2 downstream signal transduction pathway, is involved in the cell proliferation, cycle and apoptosis. Many studies indicate that SHP2 positively controls PI3K/AKT signaling by PTP catalytic activity. Our studies indicated the positive correlation between SHP2 phosphorylation and the PI3K/AKT signaling pathway since compound 2 restrained the phosphorylation of SHP2 and AKT in A549 cells.

SHP2 is broadly expressed in many human tissues and controls lots of cellular events which are important for bodily functions [20]. SHP2 also regulates cell differentiation, proliferation, and apoptosis, and controls some cancer cell metabolism, transfer and invasion [21]. Under normal physiological conditions, SHP2 is in a self-inhibition state. However, the self-inhibition of SHP2 is relieved under pathological conditions and results in abnormal cell proliferation. Inhibition of SHP2 by small molecule or knockdown SHP2 significantly restrains the migration and invasion of gastric, pancreatic cancer, and non-small-cell lung cancer cells [22,23,24]. However, the exact role of SHP2 in hepatic carcinoma (HCC) remains controversial. Deletion of SHP2 obviously promotes diethylnitrosamine-induced HCC progression, the down-regulation of SHP2 is also observed in human hepatocellular, and SHP2 acts as a tumor-suppressor gene [25]. However, some researchers have found that SHP2 serves as an oncogene in HCC, and promotes HCC progression, and SHP2 is therefore promising as a biomarker for the prognosis of HCC patients [26]. The diametrically opposite effect of SHP2 may be due to different research subjects. Furthermore, SHP2 knockout can obviously inhibit the progression of KRAS mutant non-small cell lung cancer (NSCLC). SHP2 expression is significantly increased in NSCLC patients, and its high expression is closely related to lymph node metastasis. Abnormally high expression of SHP2 enhances NSCLC cell proliferation, migration and invasion, and inhibition or knockdown of SHP2 impedes the A549 epithelial–mesenchymal transition (EMT) [27,28]. Our study revealed that compound **2** directly targeted SHP2 and restrained A549 cell proliferation in a concentration-dependent manner. The research also found that inhibition or deletion of SHP2 resulted in myeloma and leukemia cell apoptosis [29,30]. We found that incubation with compound **2** caused a significant increase in early and late apoptotic A549 cells, and the apoptosis-related pathways were also activated.

## 4. Materials and Methods

### 4.1. General Experimental Procedures

NMR was recorded on a Bruker-AVII-400 spectrometer with TMS as the internal standard. HRESIMS was obtained using Waters Synapt G_2_HDMS. Semipreparative HPLC was performed on an M/HPLC-52 system (Saipuruisi, Beijing, China) with an SPD-10AVP detector using a YMC-Pack ODS-A column (250 mm × 10 mm, 5 μm) at 208 nm. Silica gel (Qindao Marine Chemical Factory, 200–300 mesh) for column chromatography (CC). Precoated silica gel plates were used for TLC. Sephadex LH-20 was purchased from Pharmacia, Sweden. MCI gel (75–150 μm, Mitsubishi Chemical Corporation, Tokyo, Japan).

### 4.2. Plant Material

Whole plants of *A. ovalifolia* var. *calantha* were collected from Aba, Sichuan, China in July 2017 and identified by Doctor Wen-Ji Zhao, Institute of Botany, Sichuan Academy of Grassland Sciences. A voucher specimen (No. JGC201707) was deposited at Pharmacy College, Chengdu University of Traditional Chinese Medicine.

### 4.3. Cell Culture and Regents

The Human Non-Small Cell Lung Cancer Cells A549 were purchased from Procell Life Science and Technology (Wuhan, China), and maintained at 37 °C and 5% CO_2_ in DMEM medium supplemented with 10% FBS and 1% penicillin/streptomycin solution, respectively. The CCK-8 and Annexin V-FITC apoptosis detection Kit were obtained from Elabscience (Wuhan, China).

### 4.4. Extraction and Isolation

The air-dried and powdered (4 kg) *A. ovalifolia* var. *calantha* were extracted with 95% EtOH (3 × 30 L). After concentration under reduced pressure, the water-soluble residue was extracted with EtOAc. The EtOAc layer (200 g) was subjected to MCI (90% MeOH-H_2_O solution) and silica gel column chromatography (MeOH-CH_2_Cl_2_, 10:1 to 1:1, v/v) to generate eleven fractions (Fr.1–Fr.11). Fr.3 (1.5 g) was fractionated using Sephadex LH-20 (CH_2_Cl_2_-MeOH, 40:60, *v*/*v*) to give five subfractions (Fr.3.1–Fr.3.5). Fr.3.3 (40 mg) was purified by preparative TLC to yield compound **1** (4.8 mg) and compound **2** (5.9 mg). Fr.3.4 (70 mg) was further separated by semipreparative HPLC, then followed by Sephadex LH-20 (MeOH) to afford compound **3** (6 mg). 

### 4.5. Cell Viability Assay

When the A549 cell covered 80% of cell culture dish, these cells were seeded in 96-well plates at 5 × 10^3^ cells/well, respectively. After the cells adhered to the plates, various concentrations of compounds **1**–**3** were added in and incubated for another 72 h. Then, 10% CCK-8 reagent was added to the medium and incubated for another 2 h. The cell viability affected by compounds was reflected by the absorbance at 450 nm [31].

### 4.6. The Activity of SHP1 and SHP2 In Vitro

*Escherichia coli* BL21 was used to express SHP1 and SHP2 protein and proteins were purified with GST tag. In total, 15 nM SHP1 or SHP2 purified protein, a test compound (10 μM) or DMSO and reaction buffer in 100 μL constitute the reaction system. DiFMUP (10 μM) was added to the reaction system to initiate the reaction [12]. The inhibitory effects of compounds **1**–**3** were detected by Varioskan Lux with 355 nm excitation and 460 nm emission wavelengths.

### 4.7. Western Blot 

After compound **2** treatment, RIPA buffer was used to lyse cells’ protein. Equal sample proteins were added in SDS-PAGE, separated with 100 V in 60 min, then transferred into PVDF membranes, and the membranes incubated with corresponding primary antibodies (Huabio, Hangzhou, China) [32]. GAPDH or Tubulin served as normal control.

### 4.8. Cellular Thermal Shift Assay

A total of 549 cells in 12 cm dish were collected and washed with PBS three times. After three rounds of liquid nitrogen-thaw and centrifugation at 13,000× *g* for 10 min at 4 °C, the cell supernatant was collected. The cell supernatant was divided into two aliquots of 50 μL each. Compound **2** or DMSO was added to each sample. The sample was heated from 50 °C to 62 °C, then cooled for 3 min [33]. Finally, all samples were processed under 4.7.

### 4.9. Molecular Docking

Molecular docking of compound 2 was performed using crystal structure of SHP2 (PDB ID: 5EHR) from RSCB protein data bank (http://www.rcsb.org, accessed on 26 August 2021). The structure of compound **2** was drawn using the software Chem 3D 16.0 and was optimized for energy and geometry using MMFF94 force field. 5EHR and compound 2 were prepared by AutoDockTools (version 1.5.6, Molecular Graphics Lab, San Francisco, CA, USA) by removing water, adding polar hydrogens and computing Gasteiger charges. Later, a grid box with an area of 16 Å^3^ was established for the SHP2 docking site and was centered toward the coordinates of (22.586, 41.238, 5.392) where the original binding ligand SHP099 (coded as 5OD) was situated. The virtual docking was implemented in the AutoDock Vina (version 1.1.2, Molecular Graphics Lab, CA, USA) and the best docking pose was predicated based on the docked free energy and inhibition constant. The interaction between compound 2 and 5EHR was analyzed by PLIP (https://plip-tool.biotec.tu-dresden.de/plip-web/plip/index, accessed on 10 August 2022) and LigPlus software (version 2.2, European Bioinformatics Institute, Cambridge, UK). The 3D binding model was visualized by Pymol (version 1.7.2, Schrödinger, LLC, New York, NY, USA) [34]. 

### 4.10. Pharmacokinetic Properties Prediction

The pharmacokinetic properties reflect the drug absorption, distribution, metabolism and excretion. To evaluate the pharmacokinetic properties of compound **2**, the SwissADME (http://www.swissadme.ch, accessed on 10 August 2022) was used in this study. The SwissADME platform is a web-based free tool that can predict the pharmacokinetic properties of small molecules, including the pharmacokinetic properties of lipophilicity, water solubility, drug-likeness, and medicinal chemistry.

### 4.11. A549 Cell Wound-Healing Assay 

When the A549 cell covered 80% of cell culture dish, these cells were seeded in 6-well plates at 5 × 10^5^ cells/well, respectively. After the cells adhered to the plates, wound formation was performed by 200 μL pipette tips. Then, A549 cells were treated with different concentrations of compound **2** for 72 h. The image of each gap was obtained by Leica TCS SP8 (Leica, Wetzlar, Germany) at 0, 24, 72 h, respectively [35].

### 4.12. A549 Cell Invasion Assay

The inner chamber was pre-coated with matrigel. Cells were seeded in the inner chamber with FBS-free medium, and DMEM medium with 5% FBS was added to the outer chamber. After a 24 h treatment with compound **2**, 4% paraformaldehyde was used to fix the cells and 0.1% crystal violet was used to stain A549 cells. The migrating cells were observed with a microscope [34].

### 4.13. Apoptosis Flow Cytometry

After 72 h treatment with different concentrations of compound **2**, A549 cells were collected and rinsed three times with PBS. A 195 μL Annexin V-FITC buffer, 5 μL Annexin V-FITC, and 10 μL PI were sequentially added to each sample. The apoptotic cells were detected and analyzed by flow cytometry (ACEA NovoCyte, Hangzhou, China) [12].

### 4.14. Caspase-3 Activity Assay 

Ac-DEVD-AFC, a caspase-3 fluorimetric substrate, was used to detect caspase-3 activity. After 72 h treatment with compound **2**, cell supernatants were collected. The 20 μL cell supernatants, 100 μM Ac-DEVD-AFC, and reaction buffer constituted the 100 μL reaction system, and was then incubated at 37 °C for another 1 h. Caspase-3 activity was detected by Varioskan Lux with 405 nm excitation and 510 nm emission wavelengths [36].

### 4.15. Statistical Analysis

In this research, GraphPad Prism 8.0 (GraphPad, La Jolla, CA, USA) software was used for data analysis. All experiments were performed in triplicate, and data were given as means ± standard. The comparisons between different groups were carried out by one-way analysis of variance. A two-sided value of *p* < 0.05 or *p* < 0.01 were regarded as statistically significant.

## 5. Conclusions

Taken together, three abietane diterpenoids, compounds **1**–**3**, were isolated from *Ajuga ovalifolia* var. *calantha.* The enzymatic study indicated that compound **2** directly targeted the SHP2 catalytic domain through hydrogen bonding and hydrophobic action. After incubation for 72 h, compound **2** significantly restrained A549 cell proliferation and invasion, and further resulted in cell apoptosis by the inhibition of the SHP2–ERK/AKT pathways.

## Data Availability

The data that support the findings of this study are available from the corresponding author upon reasonable request.

## Supplementary Materials

The following supporting information can be downloaded at: https://www.mdpi.com/article/10.3390/molecules27175469/s1, Figures S1–S8: The HRESI (+)MS, 1H NMR, and 13C NMR spectra of compounds 1–3; Figure S9: The effects of compound 2 on cellular SHP2/p-SHP2 expression and cellular thermal shift assay between SHP2 and different doses of compound 2; Figure S10: Molecular docking of SHP099 with SHP2; Figures S11–S16: The raw results of molecular docking from Autodock Vina, PLIP and LigPlus; Figure S17: The relative expression of p-ERK, p-AKT and p-STAT3 after 72 h compound 2 treatment; Figure S18: The relative expression of cleaved caspase 8,9,3 and cleaved PARP after 72 h compound 2 treatment; Table S1: List of bonding interactions of SHP099 and compound 2 binding to SHP2; Table S2: List of pharmacokinetic properties of compound 2.
