# Supplementary material for: Ajuforrestin A, an Abietane Diterpenoid from Ajuga ovalifolia var. calanthe, Induces A549 Cell Apoptosis by Targeting SHP2
Source: Molecules. 2022 Aug 25;27(17):5469. doi: 10.3390/molecules27175469 (PMC9457730; doi:10.3390/molecules27175469)
Supplement: Supplementary file 1 [file molecules-27-05469-s001.zip › molecules-1849923-supplementary.pdf]

List of supporting information

Figure S1. ^{1}H NMR (400 MHz, $CDCl_3$) spectrum of **1**

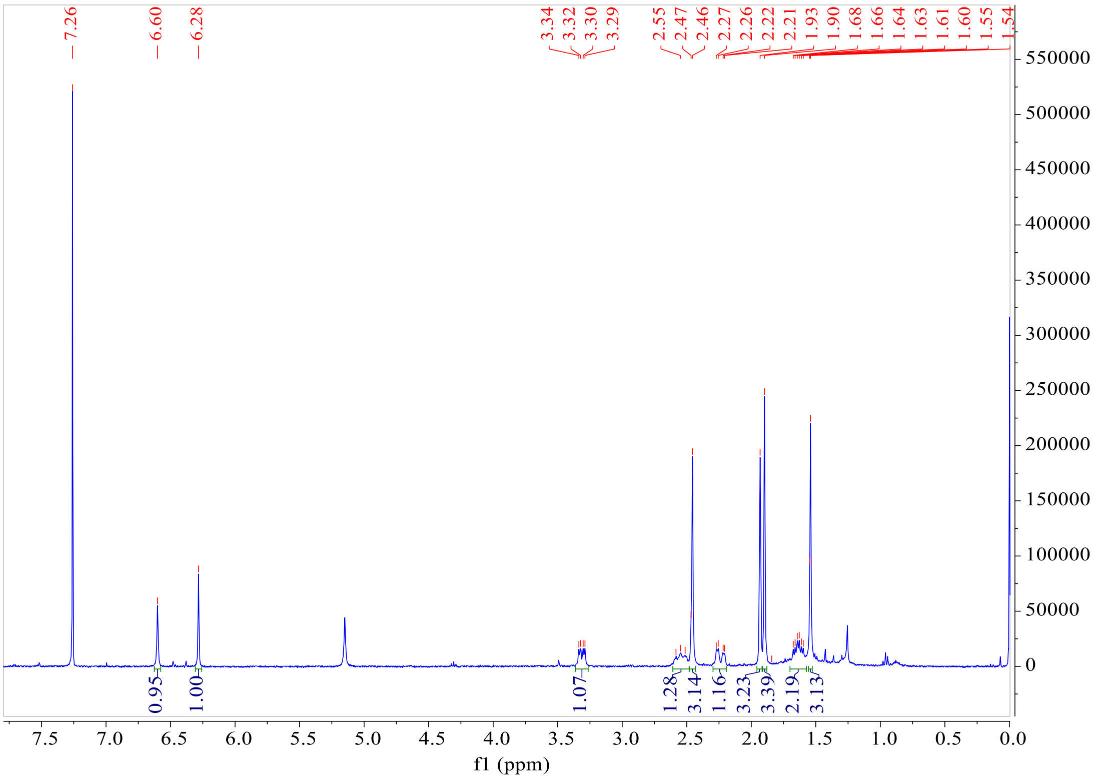

Figure S2. ^{13}C NMR (100 MHz, $CDCl_3$) spectrum of **1**

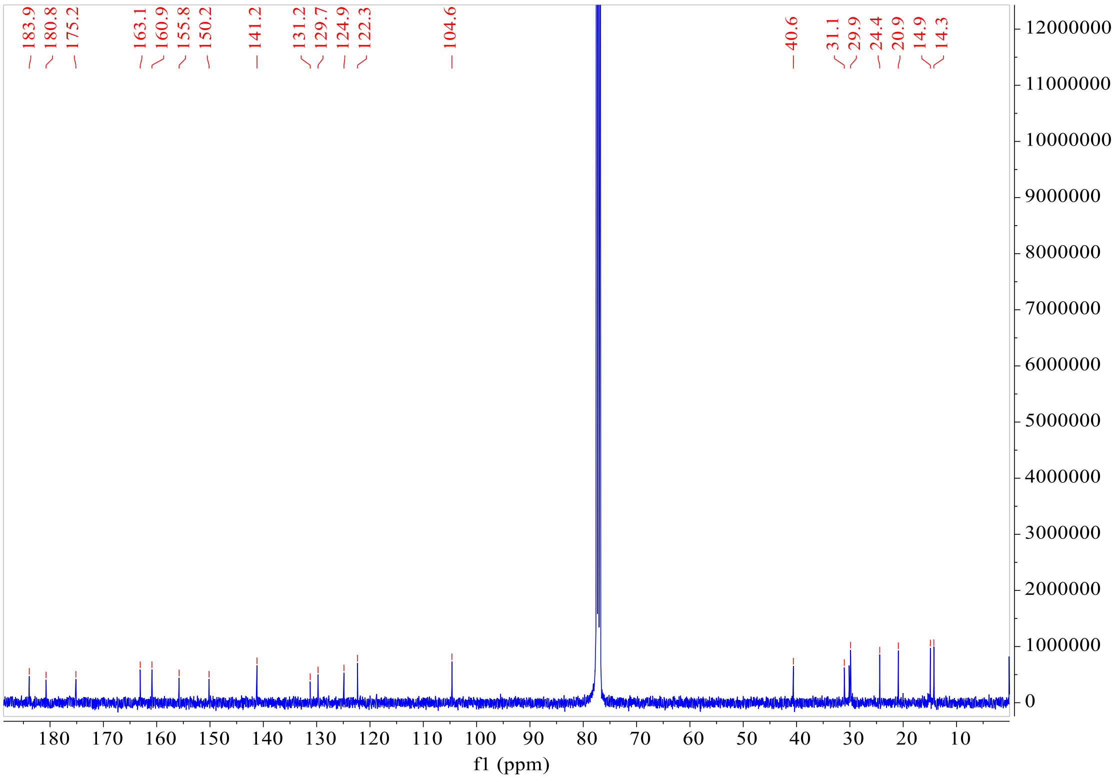

Figure S3. HRESI (+) MS spectrum of **2**

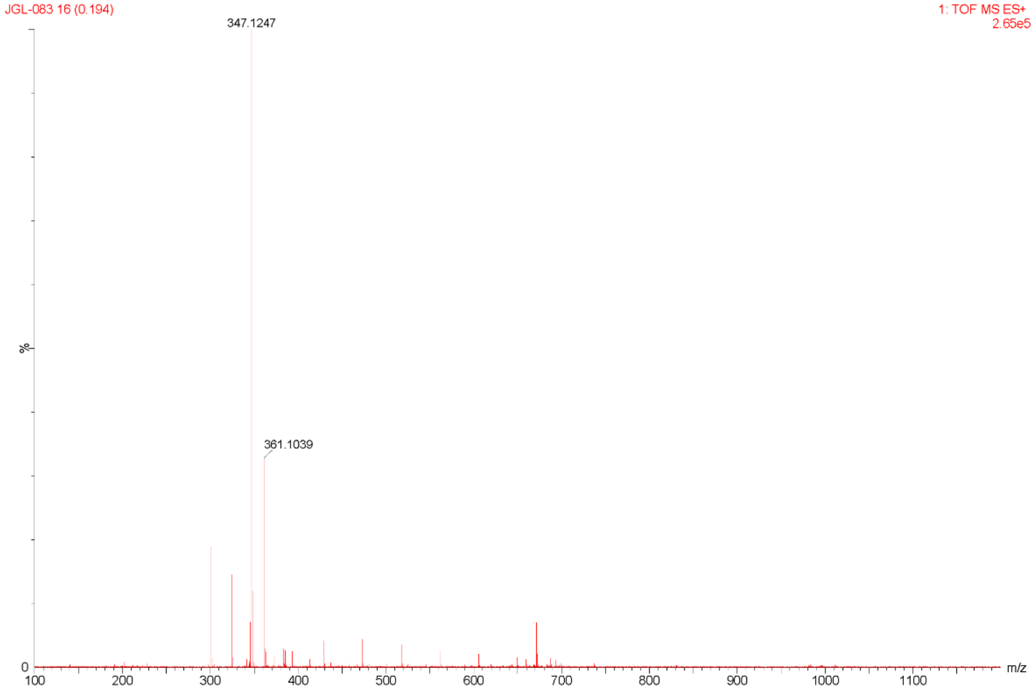

Figure S4. ^{1}H NMR (400 MHz, Acetone-d_6) spectrum of **2**

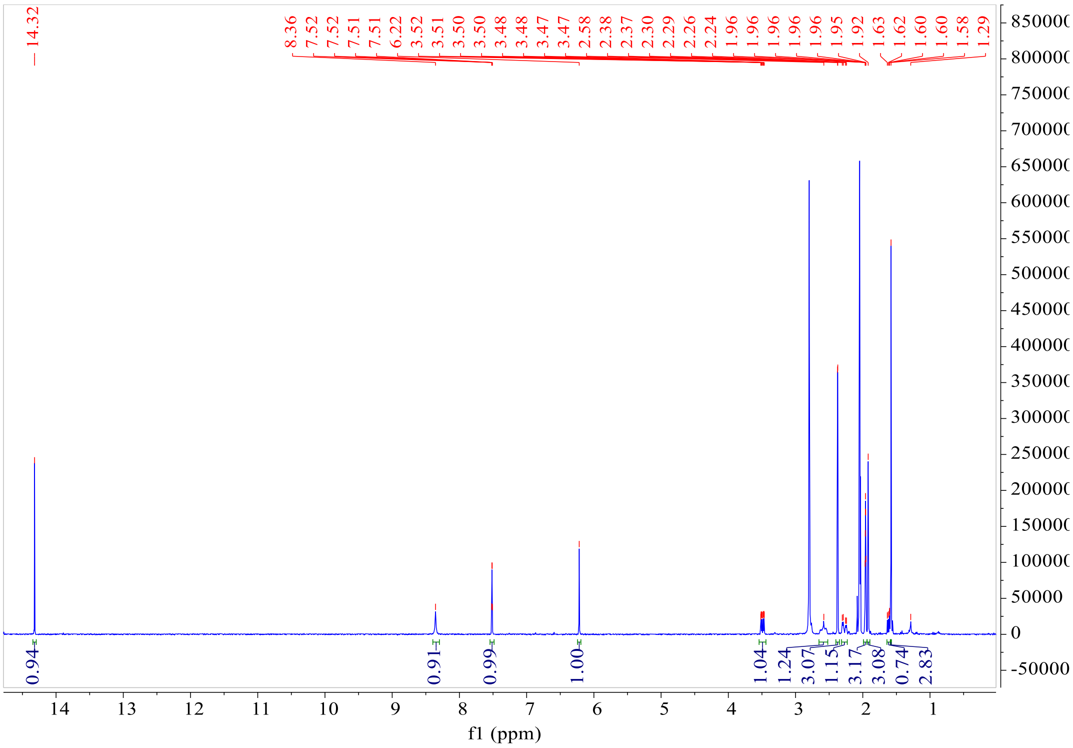

Figure S5. ^{13}C NMR (100 MHz, Acetone-d_6) spectrum of **2**

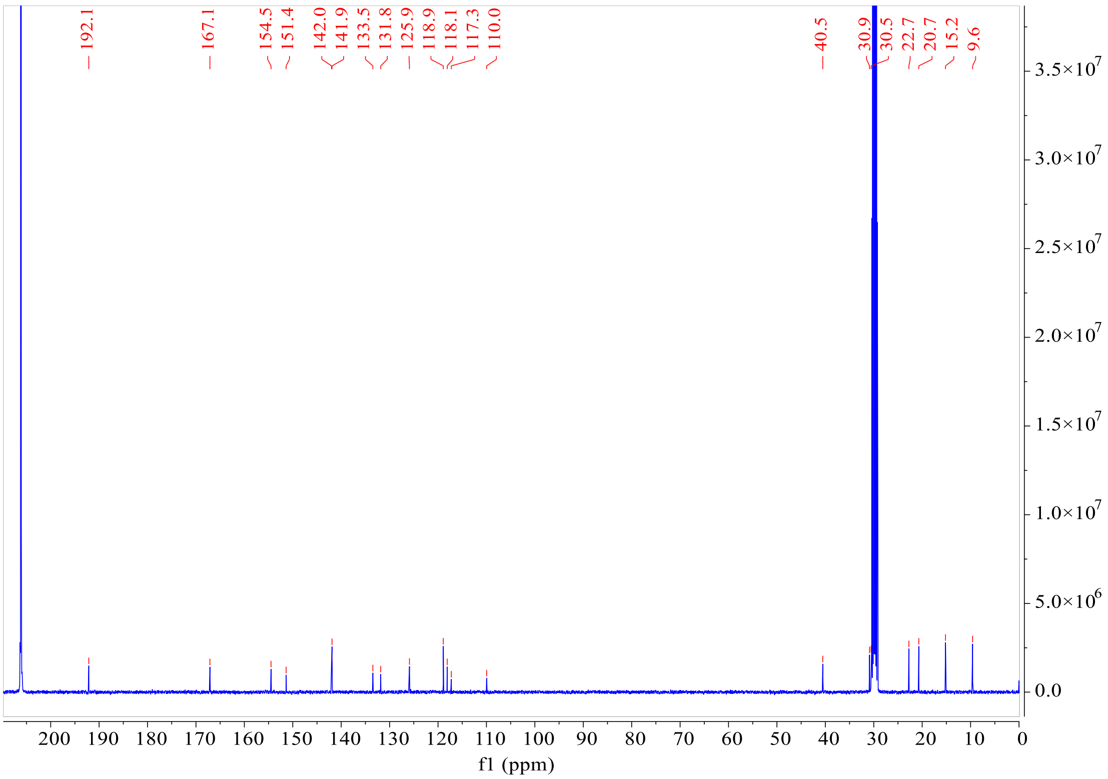

Figure S6. HRESI (+) MS spectrum of **3**

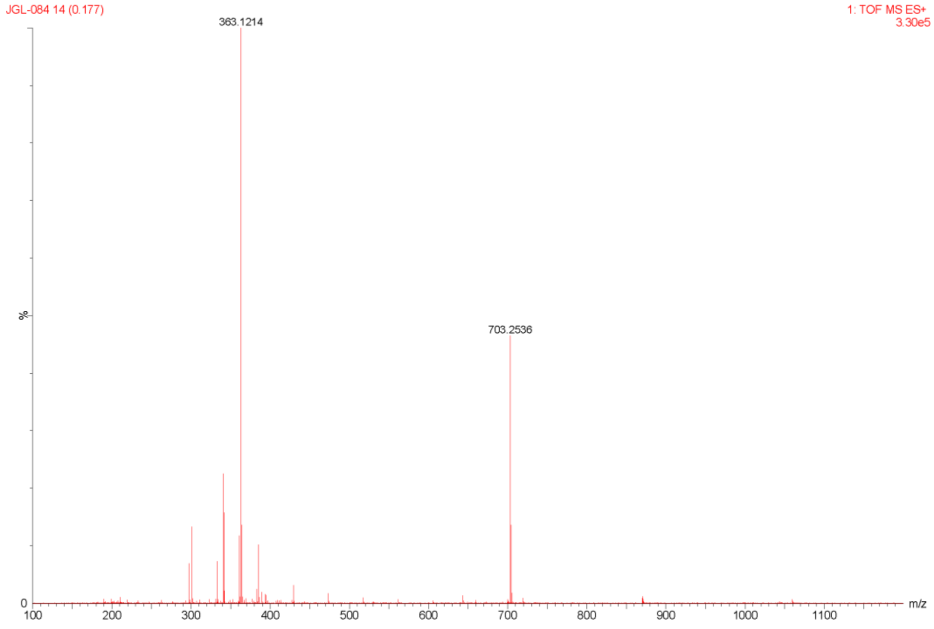

Figure S7. ^{1}H NMR (400 MHz, $CDCl_3$) spectrum of **3**

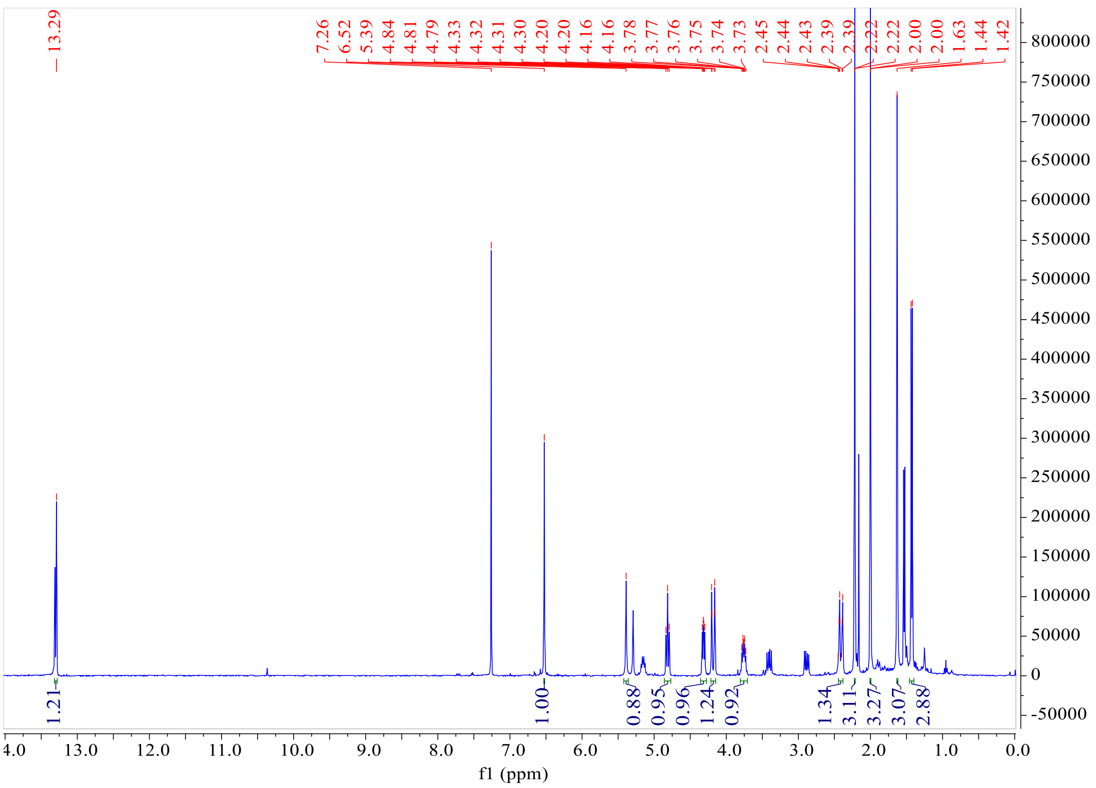

Figure S8. ^{13}C NMR (100 MHz, $CDCl_3$) spectrum of **3**

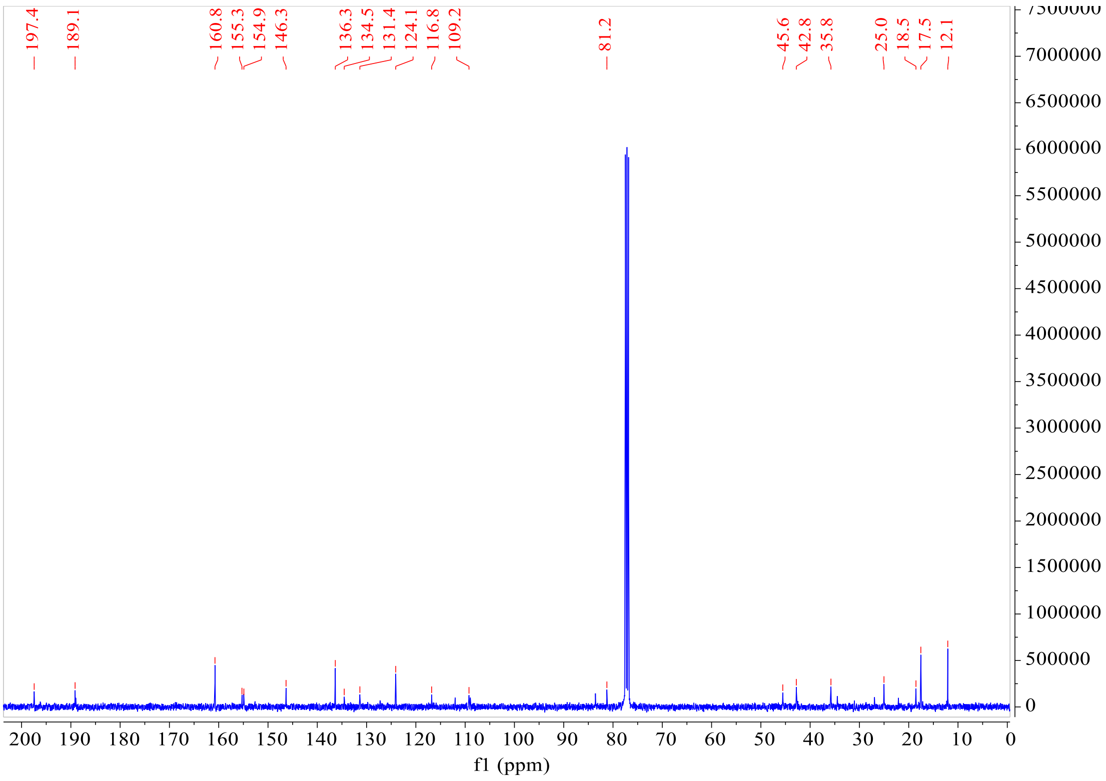

Figure S9. **(A, B)**The effects of compound **2** on cellular SHP2/p-SHP2 expression.

(C) Cellular thermal shift assay between SHP2 and different dose compound **2**.

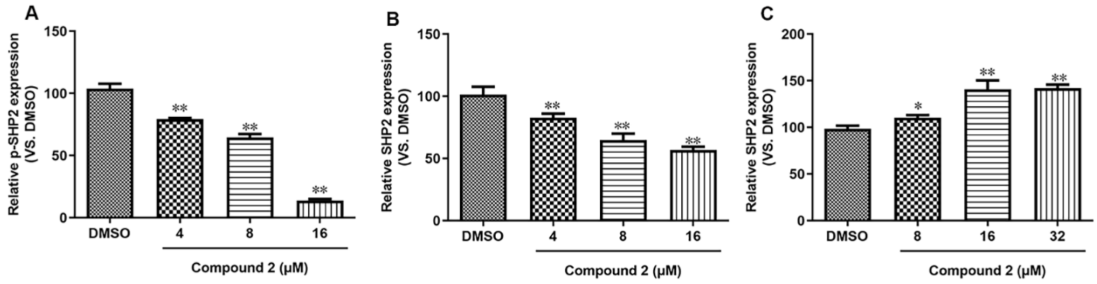

Figure S10. Molecular docking of SHP099 with SHP2 (PDB ID: 5EHR, green dash, hydrogen bonds; yellow dash, π-cation interaction; purple dash, halogen bond; other residues, hydrophobic interactions)

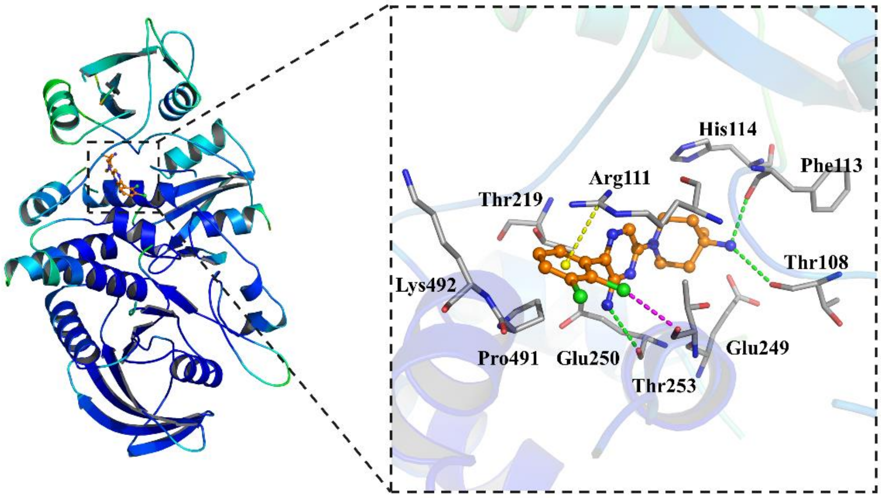

Figure S11. Docking pose and affinity energy of compound 2 binding to SHP2 (PDB ID: 5EHR)

mode	affinity (kcal/mol)	dist from best mode rmsd l.b.	rmsd u.b.
1	-7.8	0.000	0.000
2	-7.6	2.492	6.095
3	-6.6	1.751	2.890
4	-6.5	3.859	6.203
5	-6.2	1.944	6.519
6	-5.9	4.057	6.126
7	-5.8	3.828	7.268
8	-5.8	1.571	3.171
9	-5.5	3.908	7.477

Figure S12. Docking pose and affinity energy of SHP099 binding to SHP2 (PDB ID: 5EHR)

mode	affinity (kcal/mol)	dist from best mode rmsd l.b.	rmsd u.b.
1	-10.9	0.000	0.000
2	-10.2	1.381	1.794
3	-9.1	2.121	3.059
4	-8.5	1.423	2.327
5	-8.2	4.516	8.918
6	-8.0	4.272	8.692

Figure S13. Docking analysis of compound **2** binding to SHP2 (PDB ID: 5EHR) by PIPL

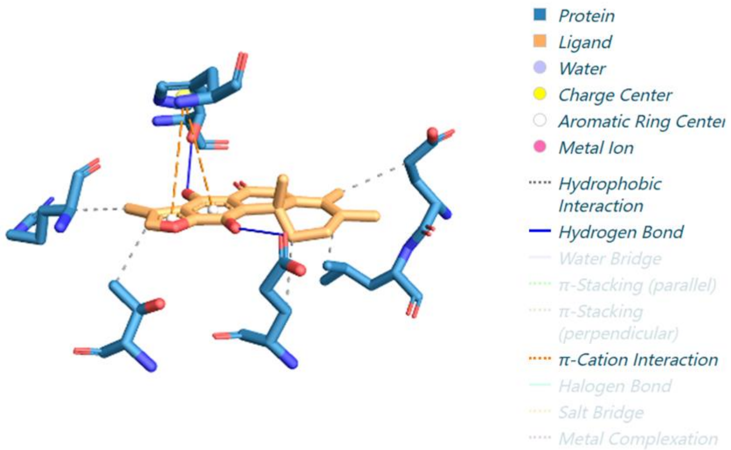

Hydrophobic Interactions

Index	Residue	AA	Distance	Ligand Atom	Protein Atom
1	111A	ARG	3.91	7714	1517
2	232A	GLU	3.68	7712	3118
3	233A	LEU	3.49	7712	3135
4	249A	GLU	3.90	7708	3307
5	253A	THR	3.89	7705	3373

Hydrogen Bonds

Index	Residue	AA	Distance H-A	Distance D-A	Donor Angle	Protein donor?	Side chain	Donor Atom	Acceptor Atom
1	218A	THR	1.69	2.59	153.12	×	✔	7730 [O3]	2890 [O3]
2	249A	GLU	2.31	2.69	102.42	×	✔	7729 [O3]	3310 [O2]

π-Cation Interactions

Index	Residue	AA	Distance	Offset	Protein charged?	Ligand Group	Ligand Atoms
1	114A	HIS	5.35	1.16	✔	Aromatic	7705, 7717, 7722, 7723, 7728
2	114A	HIS	5.30	1.01	✔	Aromatic	7707, 7717, 7719, 7720, 7721, 7723

Figure S14. Docking analysis of compound **2** binding to SHP2 (PDB ID: 5EHR) by LigPlus

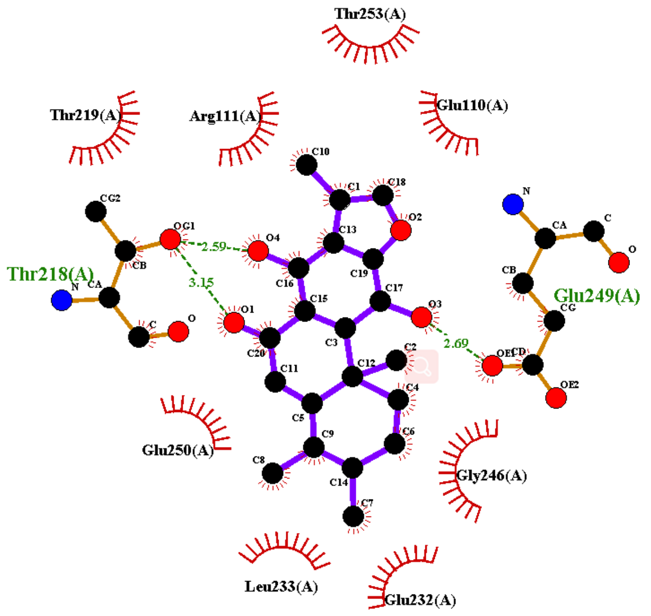

Figure S15. Docking analysis of SHP099 binding to SHP2 (PDB ID: 5EHR) by PIPL

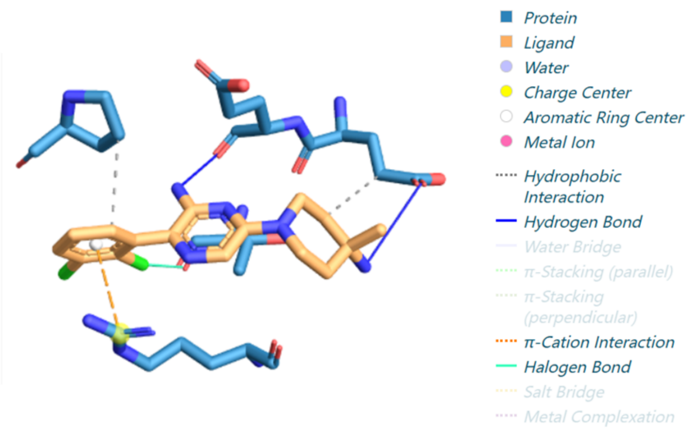

Index	Residue	AA	Distance	Ligand Atom	Protein Atom
1	249A	GLU	3.85	7717	3308
2	491A	PRO	3.68	7725	7112

Hydrogen Bonds

Index	Residue	AA	Distance H-A	Distance D-A	Donor Angle	Protein donor?	Side chain	Donor Atom	Acceptor Atom
1	249A	GLU	3.02	3.89	138.98	×	✓	7726 [N3]	3310 [O2]
2	250A	GLU	2.11	3.10	152.64	×	×	7713 [Npl]	3321 [O2]

π-Cation Interactions

Index	Residue	AA	Distance	Offset	Protein charged?	Ligand Group	Ligand Atoms
1	111A	ARG	3.60	0.64	✓	Aromatic	7714, 7721, 7722, 7723, 7724, 7725

Halogen Bonds

Index	Residue	AA	Distance	Donor Angle	Acceptor Angle	Donor Atom	Acceptor Atom
1	253A	THR	3.30	165.68	92.66	7705 [Cl]	3371 [O2]

Figure S16. Docking analysis of SHP099 binding to SHP2 (PDB ID: 5EHR) by LigPlus

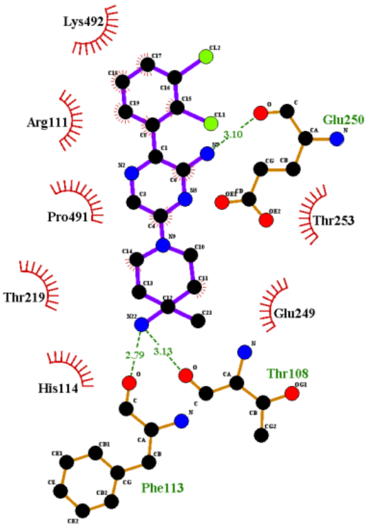

Figure S17. The relative expression of p-ERK, p-AKT and p-STAT3 after 72 h compound 2 treatment.

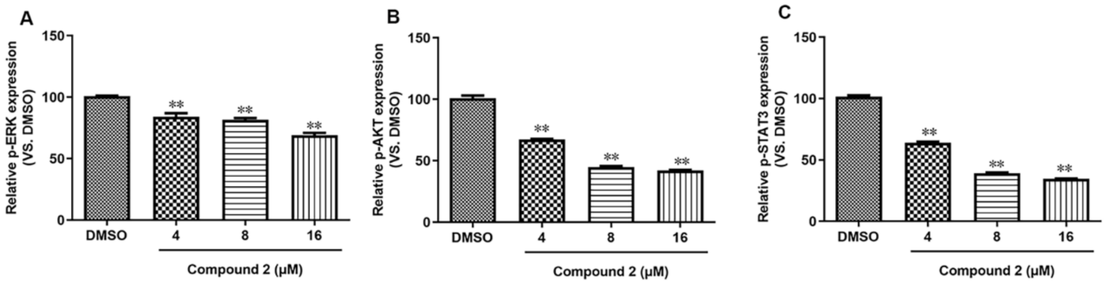

Figure S18. The relative expression of cleaved caspase 8,9,3 and cleaved PARP after 72 h compound 2 treatment.

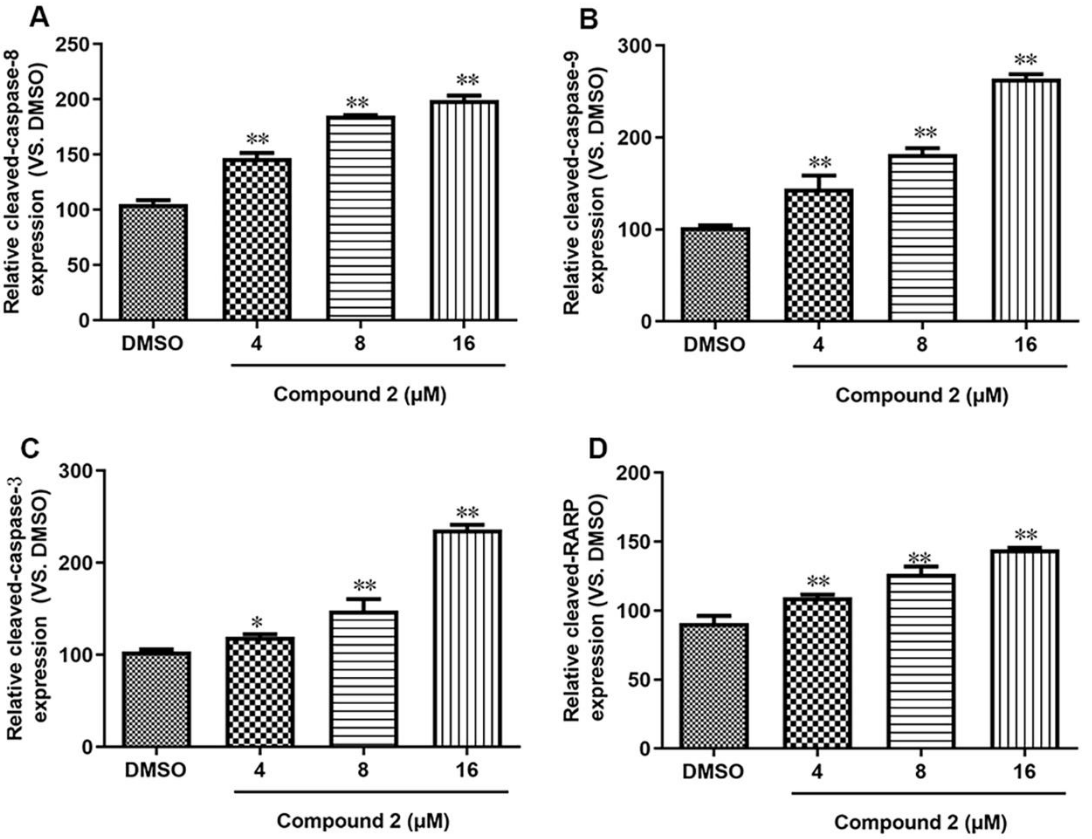

Table S1. List of bonding interactions of SHP099 and compound **2** binding to SHP2 (PDB ID: 5EHR)

Compounds	Residues	Distance (Å)	Bond type
SHP099	Glu250	3.10	Hydrogen bonds

	Thr108	3.13	Hydrogen bonds
	Phe113	2.79	Hydrogen bonds
	Thr253	3.30	Halogen bonds
	Arg111	3.60	π-cation interactions
	Lys492	< 4	Hydrophobic interactions
	Arg111	< 4	Hydrophobic interactions
	Pro491	< 4	Hydrophobic interactions
	Thr219	< 4	Hydrophobic interactions
	His114	< 4	Hydrophobic interactions
	Thr253	< 4	Hydrophobic interactions
	Glu249	< 4	Hydrophobic interactions
Compound **2**	THR218	2.59	Hydrogen bonds
	THR218	3.15	Hydrogen bonds
	GLU249	2.69	Hydrogen bonds
	His114	5.30	π-cation interactions
	His114	5.35	π-cation interactions
	Clu110	< 4	Hydrophobic interactions
	Arg111	< 4	Hydrophobic interactions
	Glu249	< 4	Hydrophobic interactions
	Gly246	< 4	Hydrophobic interactions
	Leu233	< 4	Hydrophobic interactions
	Glu250	< 4	Hydrophobic interactions
	Glu232	< 4	Hydrophobic interactions
	Thr219	< 4	Hydrophobic interactions
	Thr253	< 4	Hydrophobic interactions

Table S2. List of pharmacokinetics properties of compound **2**, including pharmacokinetic properties, lipophilicity, water solubility, drug-likeness, and medicinal chemistry.

Properties		Compound **2**
Physicochemical	MW (g/mol)	324.37 g/mol
Properties	Heavy atoms	24
	Arom. Heavy atmos	9
	Rotatable bonds	0
	H-bond acceptors	4
	H-bond donors	2
	TPSA (≤ 140 Å^2)	70.67 Å^2
Lipophilicity	Consensus Log $P_{o/w}$	3.74
Water solubility	Log S (ESOL)	Moderate
Pharmacokinetics	GI absorption	High
	BBB permeant	Yes
Drug-likeness	Lipinski	Yes
	Bioavailability score	0.55
Medi. Chemistry	PAINS	1 alert

Synth. accessibility	4.16
